# Perspectives of African stakeholders on gene drives for malaria control and elimination: a multi-country survey

**DOI:** 10.1186/s12936-023-04787-w

**Published:** 2023-12-21

**Authors:** Marceline F. Finda, Elijah O. Juma, Najat F. Kahamba, Rhosheen S. Mthawanji, Maganga Sambo, Basiliana Emidi, Susan Wiener, David O’Brochta, Michael Santos, Stephanie James, Fredros O. Okumu

**Affiliations:** 1 Environmental Health and Ecological Sciences, Ifakara Health Institute, PO Box 53, Ifakara, Tanzania; 2 Pan-African Mosquito Control Association (PAMCA), Off Mbagathi Road, PO Box 44455-00100, Nairobi, Kenya; 3 Malawi Liverpool Wellcome Trust Clinical Research Programme, Blantyre 3, PO Box 30096, Chichiri, Malawi; 4 National Institute for Medical Research, PO Box 1462, Mwanza, Tanzania; 5 Foundation for the National Institutes of Health, 11400 Rockville Pike, Suite 600, North Bethesda, MD 20852 USA; 6 School of Life Science and Bioengineering, The Nelson Mandela African Institution of Science and Technology, P. O. Box 447, Arusha, Tanzania; 7 School of Biodiversity, One Health and Veterinary Medicine, University of Glasgow, Glasgow, G128QQ UK; 8 School of Public Health, Faculty of Health Sciences, University of the Witwatersrand, 1 Smuts Avenue, Braamofontein, 2000 South Africa

**Keywords:** Stakeholder perspectives, Gene drive modified mosquitoes, Malaria elimination, Africa

## Abstract

**Background:**

Gene drive modified mosquitoes (GDMMs) have the potential to address Africa’s persistent malaria problem, but are still in early stages of development and testing. Continuous engagement of African stakeholders is crucial for successful evaluation and implementation of these technologies. The aim of this multi-country study was, therefore, to explore the insights and recommendations of key stakeholders across Africa on the potential of GDMMs for malaria control and elimination in the continent.

**Methods:**

A concurrent mixed-methods study design was used, involving a structured survey administered to 180 stakeholders in 25 countries in sub-Saharan Africa, followed by 18 in-depth discussions with selected groups and individuals. Stakeholders were drawn from academia, research and regulatory institutions, government ministries of health and environment, media and advocacy groups. Thematic content analysis was used to identify key topics from the in-depth discussions, and descriptive analysis was done to summarize information from the survey data.

**Results:**

Despite high levels of awareness of GDMMs among the stakeholders (76.7%), there was a relatively low-level of understanding of their key attributes and potential for malaria control (28.3%). When more information about GDMMs was provided to the stakeholders, they readily discussed their insights and concerns, and offered several recommendations to ensure successful research and implementation of the technology. These included: (i) increasing relevant technical expertise within Africa, (ii) generating local evidence on safety, applicability, and effectiveness of GDMMs, and (iii) developing country-specific regulations for safe and effective governance of GDMMs. A majority of the respondents (92.9%) stated that they would support field trials or implementation of GDMMs in their respective countries. This study also identified significant misconceptions regarding the phase of GDMM testing in Africa, as several participants incorrectly asserted that GDMMs were already present in Africa, either within laboratories or released into the field.

**Conclusion:**

Incorporating views and recommendations of African stakeholders in the ongoing research and development of GDMMs is crucial for instilling stakeholder confidence on their potential application. These findings will enable improved planning for GDMMs in Africa as well as improved target product profiles for the technologies to maximize their potential for solving Africa’s enduring malaria challenge.

## Background

Malaria control efforts, notably the use of insecticide-treated nets (ITNs), indoor residual sprays (IRS), and improved case management [1, 2], have prevented approximately 11.7 million deaths and 2 billion cases worldwide over the past two decades [3]. Unfortunately, there are still an estimated 247 million malaria cases and 619,000 malaria deaths annually, more than 95% of these in sub-Saharan Africa (SSA) [1]. Several challenges such as drug and insecticide resistance, inadequate access to prevention tools and sub-optimal use compliance have slowed the progress, and many countries have recently been reporting upsurges in malaria cases and deaths [4, 5]. Underpinning these are the weak health systems and poor socio-economic situation in malaria-endemic areas. These challenges highlight the urgent need for novel strategies to accelerate malaria elimination efforts [2].

Genetic bio-control tools, such as gene drive modified mosquitoes (GDMMs), have emerged as promising new tools that could be deployed within an integrated vector management system to improve malaria control and elimination prospects [6–8]. These technologies are rapidly gaining interest, due to their potential to overcome many of the major challenges of current malaria control tools and strategies [6–8]. GDMMs are genetically modified mosquitoes that have an additional mechanism that biases the inheritance of a particular gene, enhancing its probability of passing on to offspring [9–11]. This in turn ensures a spread of specific genetic modifications throughout the mosquito population [10, 11]. Two approaches of GDMMs are being considered, namely population suppression, which aims to significantly reduce and potentially eliminate populations of certain vector species [12], and population replacement, which aims to introduce novel genetic constructs that block disease transmission by a vector species [10, 11].

Despite extensive conversations on the potential utility of GDMMs for malaria control over the past decade, much of the available evidence is of discussions in the United States and Europe, where the initial phases of the technologies occurred [13]. Comparatively, there has been limited evidence of multi-faceted engagement on the utility of GDMMs among African stakeholders, who are the envisaged primary beneficiaries of the technologies. Although there have been efforts to raise knowledge and awareness for this technology within Africa, most of the efforts have mostly targeted specific stakeholder groups such as scientists and in some cases regulators; there has been limited wide-coverage of GDMMs to capture attention of broader African stakeholders. To ensure ethical appropriateness, and effective utilization of GDMMs in addressing malaria control and elimination in specific African countries, it is essential to incorporate, from the outset, insights, perspectives and recommendations of African stakeholders throughout the technologies’ research and development process [14–16]. Understanding the safety and efficacy characteristics of GDMMs that would be most valued by its potential end users will be fundamental to the ultimate success of these technologies as useful malaria control tools [17]. African stakeholders can provide valuable insights on social, cultural, and environmental factors, as well as local knowledge of malaria transmission dynamics. Involving them from the early stages of research and development is also critical to ensure that the technologies developed are tailored to the specific needs of the targeted sites [16, 18–21], and can minimize potential delays and backlash, as previously observed in similar technologies in India [22], Burkina Faso [23, 24] and the Cayman Islands [25].

The aim of this multi-country study was, therefore, to document insights and recommendations of key African stakeholders, regarding factors crucial to ensuring effective and impactful research and implementation of GDMMs for malaria control and elimination in Africa. Stakeholders were recruited from groups whose job responsibilities interface directly or tangentially with bio-control technologies and were considered valuable to providing divergent perspectives on gene drive technologies for malaria control. These included ministries of health, agriculture and environment, regulatory agencies, research and academic institutions, civil societies and advocacy groups and mass media groups.

## Methods

### Study design and study sites

**Fig. 1:**
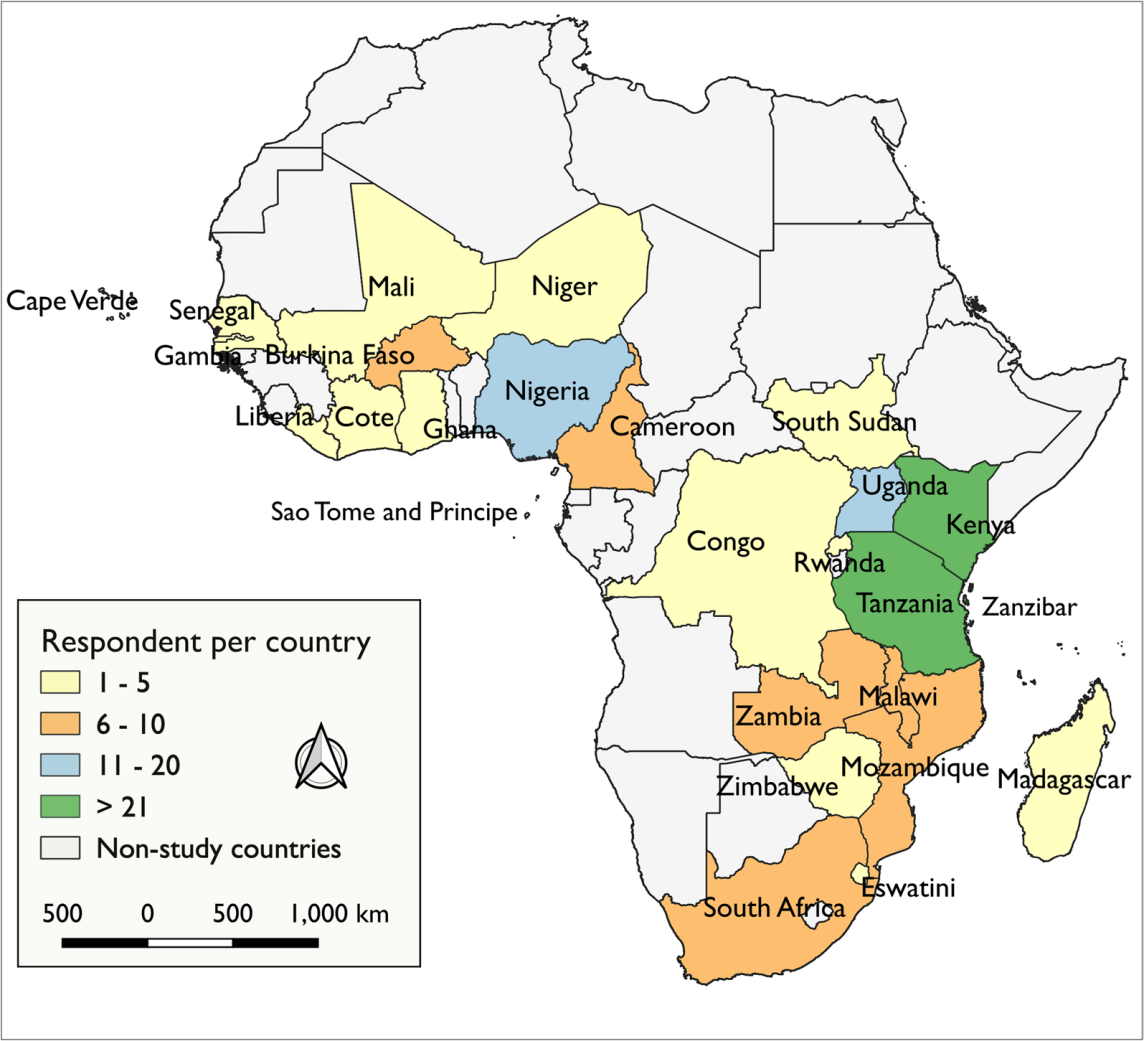
Countries represented in the study, and the corresponding number of stakeholders involved

This study used a concurrent mixed-methods design [26], which involved a survey questionnaire followed by in-depth discussions. Study participants were recruited from 25 countries in sub-Saharan Africa, including four islands (Fig. 1). The selected countries were in different geographical regions of the continent and included countries in malaria control phase as well as those currently pursuing elimination [27, 28]. The inclusion of island nations was informed by the proposition that geographical isolation may provide ecosystems well suited for conducting initial small-scale, ecologically confined field releases of GDMMs [29, 30]. This study was done between August 2020 and January 2022.

### Study participants

Study participants were recruited from purposively selected key stakeholder groups, comprising African professionals and leaders involved directly or tangentially in malaria control programs or in the use of bio-control technologies. The stakeholders represented: (i) implementation agencies in the ministries of health (i.e. National Malaria Control Programmes), agriculture and environment, (ii) research and academic institutions, (iii) civil society and advocacy groups, (iv) mainstream mass media outlets including both voice and print media, and (v) regulatory agencies, including biosafety authorities, and research and ethics approval boards.

The initial list of potential participants was compiled from various sources, including personal recommendations, existing contacts in different countries and publicly available attendance lists of previous health-related conferences. The prospective participants were contacted and invited to the study. Additionally, a snowball sampling technique [31] was used to expand the participant pool, as participants who consented to participate were asked to recommend others who might be interested in the study. All prospective participants were contacted via email, provided with a link to an online survey, and invited to engage in more in-depth discussions.

In Tanzania, due to the familiarity of the researchers with the country’s governance structure, participant selection followed a different approach. Institutional leaders representing each stakeholder group were contacted via email, requesting them to recommend suitable members of their staff to participate in the in-depth discussions. The appointed representatives were then contacted to seek their consent to participate.

### Baseline questionnaire surveys

A total of 367 stakeholders were contacted via email and phone calls. Consenting participants were provided with a baseline questionnaire in the form of an online survey administered using the KoboToolbox™ software [32]. The purpose of this survey was to assess their baseline knowledge and awareness regarding GDMMs and other bio-control methods for malaria control, and to gather their initial perceptions on potential risks and benefits associated with GDMMs, as well as their levels of support for research and implementation. The survey was divided into five parts; the first part collected information on respondents’ countries of origin and residence, and stakeholder groups they were based on. The second part collected information on socio-demographic information. Part three collected information on participants’ knowledge, awareness and perceptions of malaria situation in their countries, and sections four and five explored participants’ baseline knowledge, awareness and perceptions towards mosquito modification technologies and gene drives technologies, respectively. Out of the 367 stakeholders contacted, 180 consented and responded to the baseline survey (Table 1).

**Table 1:** Summary statistics of the main stakeholder groups participating in the study

Group	Academic institutions	Research institutions	Regulatory agencies	Government officials	Media and advocacy	Total
Participants contacted	98	96	73	63	37	367
Baseline survey	40	81	13	27	19	180
In-depth discussions	4	5	4	2	3	18

#### In-depth discussions

Originally planned as focus group discussions (FGDs), the qualitative component of the study had to be adapted due to complications caused by the COVID-19 pandemic and the diverse geographic locations of participants from 25 countries. Consequently, a majority of the in-depth discussions were conducted virtually via Zoom, incorporating various formats such as in-depth interviews or focus group discussions and other individual or group discussions, depending on the number of participants that joined a particular session. Participants who had given their consent were assigned to different stakeholder groups and invited to join the in-depth discussions. Altogether, 18 in-depth discussion sessions were completed (Table 1), each lasting 60 to 120 min. There was no segregation by gender or age during the discussions. The participants’ verbal consent was obtained for audio-recording purposes, and detailed notes were taken during the discussions. For Tanzania, however, the discussions were held in-person, following a traditional FGD structure. This was possible due to lifting up of COVID-19 restrictions in the country, and the fact that the project team was based in Tanzania.

A discussion guide was developed to explore perspectives of the participants on the potential risks, advantages, and recommendations concerning GDMMs) for malaria control in Africa. To provide a framework for the discussions, the guide was structured into three main sections. The first section prompted participants to share their insights on the malaria situation in their respective countries. They discussed their views on their countries’ overall progress, and existing challenges and opportunities. In the second section, participants were asked to deliberate on their general views regarding mosquito modification technologies, and in the third section, the discussions focused specifically on the concept of gene drives technologies for malaria control. In this section, the participants were first asked to explain what they knew about gene drives technologies for malaria control, and after the participants had provided their initial views, facilitators provided participants with accurate descriptions of GDMMs for malaria control, utilizing Microsoft PowerPoint slides [9], followed by a Q & A session for participants to seek clarifications. Subsequently, further in-depth discussions revolved around the participants’ perceptions of these technologies.

### Data processing and analysis

For the qualitative component, the recordings of the in-depth discussion were transcribed, and in the cases where the discussions were conducted in Swahili, they were translated into English. The transcriptions were done by RM, EJO and MFF. Verbatim transcripts of the FGDs were imported into NVIVO 12 Plus software for coding [33]. Both deductive and inductive coding methods were employed, with the discussion guide serving as the foundation for deductive codes, and supplementary codes emerging inductively from thorough transcript reviews and coding. Recurrent themes were extracted, and key themes were supported by direct quotes from the participants.

For quantitative data, R statistical software version 4.2.3 [34] was used to analyse socio-demographic characteristics of the survey respondents, and to summarize their knowledge regarding malaria situation in their respective countries, available interventions, as well as their knowledge and awareness of GDMMs. Respondents’ perceptions concerning their countries’ progress in malaria control, the need for alternative tools, and support for GDMMs were assessed using a Likert scale [35]. Direct comparisons of responses between the countries or the stakeholder groups were not done due to the obvious difference in representation between the countries and across the stakeholder groups.

## Results

### Characteristics of study participants

A total of 180 stakeholders from the 25 countries completed the online survey (Table 1), 60.5% (n = 109) of whom were males and 39.5% (n = 71) females. The highest educational attainment for most respondents was a PhD degree (48.9%, n = 88), nearly two thirds of the respondents were under 45 years of age (66.1%, n = 119), and scientists were the most represented stakeholder group (45.0%, n = 81) (Table 2). As for the in-depth discussions, a total of 18 sessions were conducted, consisting of four individual in-depth interviews, two small group discussions with 2–5 participants, and twelve focus group discussions with 6–10 participants each.

**Table 2:** Characteristics of stakeholders who responded to the baseline survey (n = 180)

Category	Variables	No. participants (%)
Sex	Male	109 (60.5%)
	Female	71 (39.5%)
Stakeholder group	Research institution	81 (45.0%)
	Academic institutions	40 (22.2%)
	Government agencies	27 (15.0%)
	Media and advocacy	19 (10.6%)
	Regulatory agencies	13 (7.2%)
Age group	25–35	50 (27.8%)
	36–45	69 (38.3%)
	46–55	50 (27.8%)
	> 56	11 (6.1%)
Education level attained	PhD	88 (48.9%)
	Msc/MPH/MBA	59 (32.8%)
	BSc/BA	27 (15.0%)
	Others	6 (3.3%)
Field of work	Research	109 (60.6%)
	Health care	29 (16.1%)
	Education	17 (9.4%)
	Communication	18 (10.0%)
	Others	7 (3.9%)

### Knowledge of participants on malaria prevalence and dominant vectors

When asked to indicate malaria prevalence in their countries for the previous year, nearly half (43.9%, n = 79) of the survey respondents selected a response that differed from the prevalence range indicated in the 2020 WHO malaria report for their countries [3]. About a fifth (19.4%, n = 35) of the respondents underestimated, 16.7% (n = 30) overestimated, and 7.8% (n = 14) reported not knowing malaria prevalence in their countries. The other half (56.1% (n = 101) had reasonable estimates of malaria prevalence in their countries (Table 3). Participants frequently mentioned *Anopheles gambiae*, *Anopheles arabiensis*, *Anopheles funestus* and *Anopheles coluzzii* as the dominant malaria vectors, although other vectors not typically considered significant in Africa were also mentioned infrequently (Table 3). Some respondents from eastern and southern Africa regions also incorrectly mentioned *An. coluzzii* as a dominant vector species in their countries.

**Table 3:** Perceptions of survey respondents on malaria situation in the countries

Category	Responses	No. participants (%)
Reported malaria prevalence in the country (2019) (n = 180)	Correct prevalence	101 (56.1%)
	Overestimated prevalence	30 (16.7%)
	Underestimated prevalence	35 (19.4%)
	Do not know	14 (7.8%)
Reported dominant malaria vectors in the country (n = 165)^a^	*Anopheles gambiae*	148 (89.7%)
	*Anopheles funestus*	123 (74.5%)
	*Anopheles arabiensis*	111 (67.3%)
	*Anopheles coluzzii*	33 (20.0%)
	*Anopheles nili*	18 (10.9%)
	*Anopheles merus*	13 (7.9%)
	*Anopheles moucheti*	11 (6.7%)
	*Anopheles melas*	7 (4.2%)
	*Anopheles ovengensis*	1 (0.6%)

^a^Percentages add to more than 100% because of multiple selections

The gaps in knowledge of the malaria trends became more apparent during the in-depth discussions, where most participants used vague terms like ‘*very big*’ or ‘*small problem*’ when describing malaria burden in their respective countries, even after being probed for clarification. Only a few of the participants could provide detailed information on country-specific malaria burden, ongoing interventions or the geographical distribution of malaria in their countries.

Study participants with in-depth knowledge of malaria trends in their respective countries were predominantly those drawn from the NMCPs or research institutions, in comparison to other stakeholder groups. These participants engaged in discussions about various aspects of malaria trends, including the stages of control implemented in their countries, changes in transmission dynamics over time, and persisting challenges. Interestingly, regardless of whether their countries were approaching elimination or still in control, these participants expressed the necessity for complementary interventions against malaria:

*“Malaria control in my country is stagnant although we are doing most of the conventional malaria control and prevention that are available. We are distributing bed nets and we are performing IRS in some areas in the country. But even in areas where there is intensified use of those vector control tools, we do not see the impact as we would expect… One of the questions that the NMCP here imposes on us is what else can be done, because we are doing everything that we know but still we are seeing a lot of deaths or either incidence or prevalence increasing.”* (Male scientist).

*“We do have a slightly different dynamic from other areas that are focusing on control, but we still need new technologies as we are approaching elimination. We definitely need new tools without a doubt. What has worked is working really well, but to get to the last mile we definitely need more tools.”* (Female scientist).

### Perceptions of current malaria control efforts

**Fig. 2:**
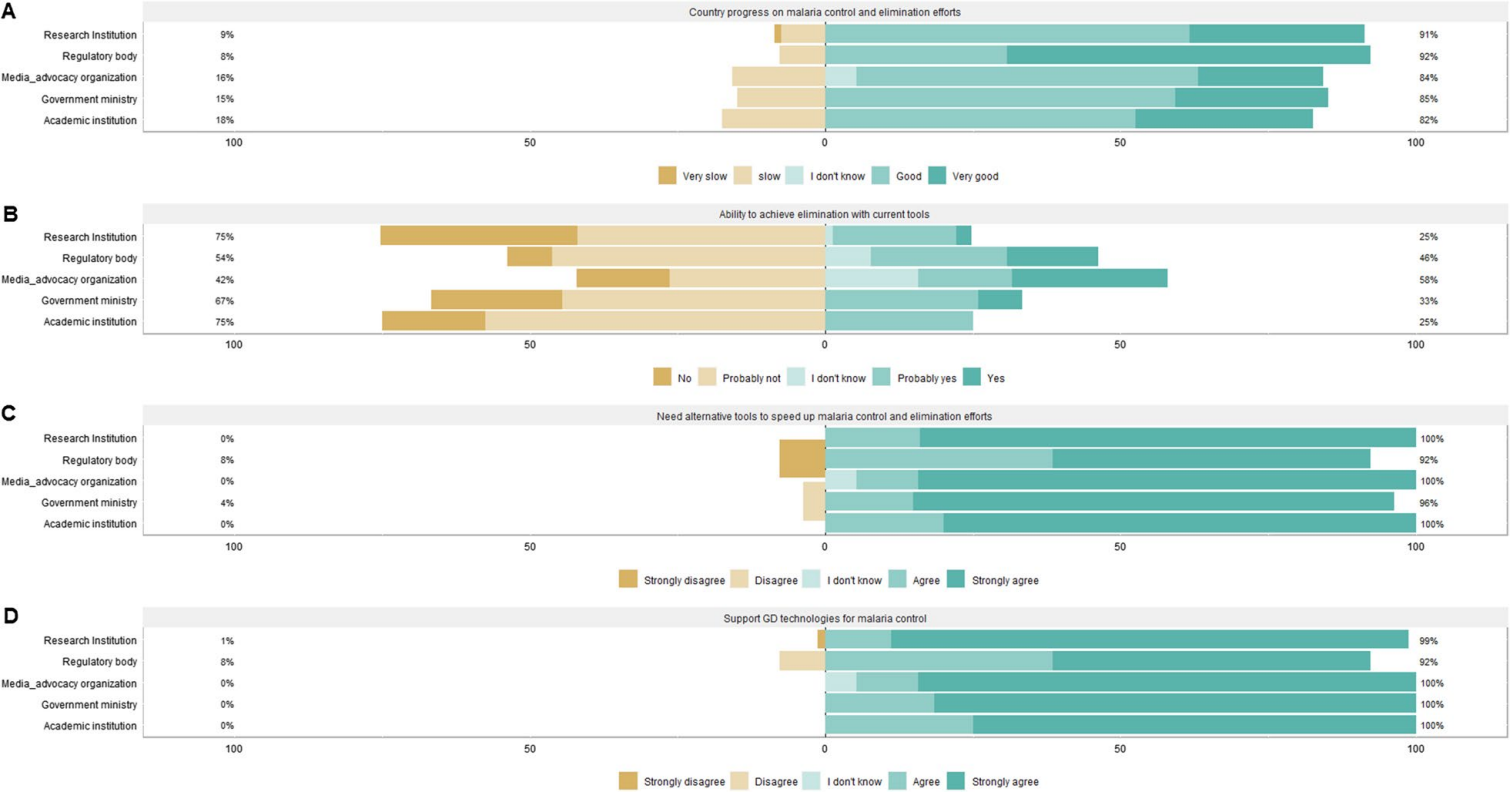
Stakeholders’ perceptions of malaria control efforts and need for alternative tools; 2A: Perceptions on the country’s progress on malaria control and elimination efforts; 2B: Perceptions on the countries’ ability to achieve malaria elimination with current efforts; 2C: Perceptions on the need for alternative tools to help speed up malaria control and elimination efforts; 2D: Current level of support for GDMMs for complementing current malaria control and elimination efforts

A majority (56.7%, n = 102) of the respondents believed that their countries were making good progress in malaria control and elimination, while 11.7% (n = 21) believed the progress was slow or stagnating (Fig. 2A). When asked if elimination could be achieved with the current tools, 44.4% (n = 80) disagreed, and 80.6% (n = 145) believed their countries would need additional tools to achieve elimination (Fig. 2B). The general perception of good progress in malaria control was also reflected in the in-depth discussions, where participants highlighted Africa’s considerable success in reducing malaria cases and deaths. A majority of the participants across the stakeholders acknowledged  the need for novel tools and strategies to complement ongoing efforts and address challenges such as insecticide and drug resistance, changes in mosquito behaviour, and the poor compliance to current malaria control interventions (Fig. 2C).

Stakeholder groups approached the discussion of progress and challenges in malaria control differently. Experts in malaria control, including scientists, academics, and NMCP representatives focused on reducing malaria cases and addressed challenges of resistance and changes in mosquito behaviour. Conversely, participants with limited knowledge in malaria control discussed progress based on their personal experience, perceived community experiences and information from the media. For example, participants from media and advocacy groups discussed their views that malaria had been largely popularized, increasing awareness of the disease and its preventive measures. These participants also highlighted community-based challenges such as difficulties in using ITNs, taking anti-malarial drugs, and fear of hospitals as these two reporters explained:

*“I think a lot of people are very aware of malaria and they are very conscious of what to do; keep the environment clean, use bed nets and insecticides. These messages are communicated everywhere these days, you cannot run away from them… but then in the rural areas, sometimes it’s not often about the environment. I think the major issue is of course, they can’t help it because they spend most of their time outdoors.”* (Female reporter).

*“In rural areas, it is actually hard to eliminate mosquitoes, and it is hard not to get malaria. The livelihoods are just not friendly for that. You know because a lot of the people take liquor, so they can’t take anti-malarial drugs. They also don’t go to the clinics or hospitals… and sometimes when they are sick, they just buy malaria drugs. Those are some of the challenges that I think keep us from eliminating malaria.”* (Female reporter).

### Awareness of gene drive modified mosquitoes (GDMMs) and ongoing work

A majority (76.7%, n = 138) of the survey participants reported having heard or read about the use of GDMMs for malaria control (Table 4). Their stated sources of information on these technologies included research articles, scientific meetings and conferences, friends and colleagues, and social media. There was a significant misconception regarding the state of GDMM testing in Africa, as numerous participants incorrectly asserted, that genuine gene drive mosquitoes were already present in Africa, either within laboratories or had already been released into the field. About one third of the participants who were aware of GDMMs claimed, sometime erroneously, that there was ongoing GDMMs research in their respective countries (Table 4). Participants from Burkina Faso, Cameroon, Cape Verde, Kenya, Liberia, Mali, Nigeria, South Africa, and Uganda claimed that there were ongoing GDMM research activities at the laboratory phase in their countries. Although no GDMMs have yet been released in Africa, a section of participants from Burkina Faso, Kenya, Mali, Nigeria, and Uganda also reported, incorrectly, that they were aware of confined releases or large-scale releases of GDMMs already going on in their countries as of the time this study (2020–2022).

**Table 4:** Awareness of survey respondents on ongoing GDMM research in countries

Category	Responses	n (%)
Aware of GDMMs (n = 180)	Yes	138 (76.7%)
	No	42 (23.3%)
Aware of ongoing GDMM research (n = 138)	Yes	44 (31.9%)
	No	94 (68.1%)
Type of GDMM technology researched in the country (n = 44)^a^	Population suppression	41 (93.2%)
	Population replacement	22 (50.0%)
	Don’t know	2 (4.5%)
Stage of ongoing GDMM research in the country (n = 44)^a^	Laboratory	34 (77.3%)
	Community stakeholder engagement	18 (40.9%)
	Confined releases	8 (18.2%)
	Large scale releases	1 (2.3%)
	Don’t know	7 (15.9%)

^a^Percentages add to more than 100% because of multiple selections

### Knowledge of GDMMs and their mechanisms in malaria control

When asked about the mechanisms of GDMMs, only 28.3% (n = 51) of the survey respondents stated that they knew how GDMMs work in malaria control. These were affiliated with research institutions (60.8%, n = 31), academic institutions (23.5% = 12), NMCPs (11.8%, n = 6) and or regulatory agencies (3.9%, n = 2). There were no significant differences in GDMM knowledge by age, educational levels or country of origin.

The differences in knowledge were also evident in the in-depth discussions, where a majority of the participants struggled to differentiate between GDMMs and other mosquito modification or bio-control technologies. When prompted to explain their understanding of GDMMs, participants often described characteristics of other mosquito modification approaches such as Sterile Insect technique (SIT) [36–38], non-gene drives genetically modified mosquito technologies [39], or in some cases *Wolbachia*-modified mosquitoes currently being used for control of *Aedes*-borne diseases [40, 41]. For further discussions about GDMMs however, the facilitators provided participants with correct descriptions of the technologies.

### Perceptions of GDMMs for malaria control and elimination

**Fig. 3:**
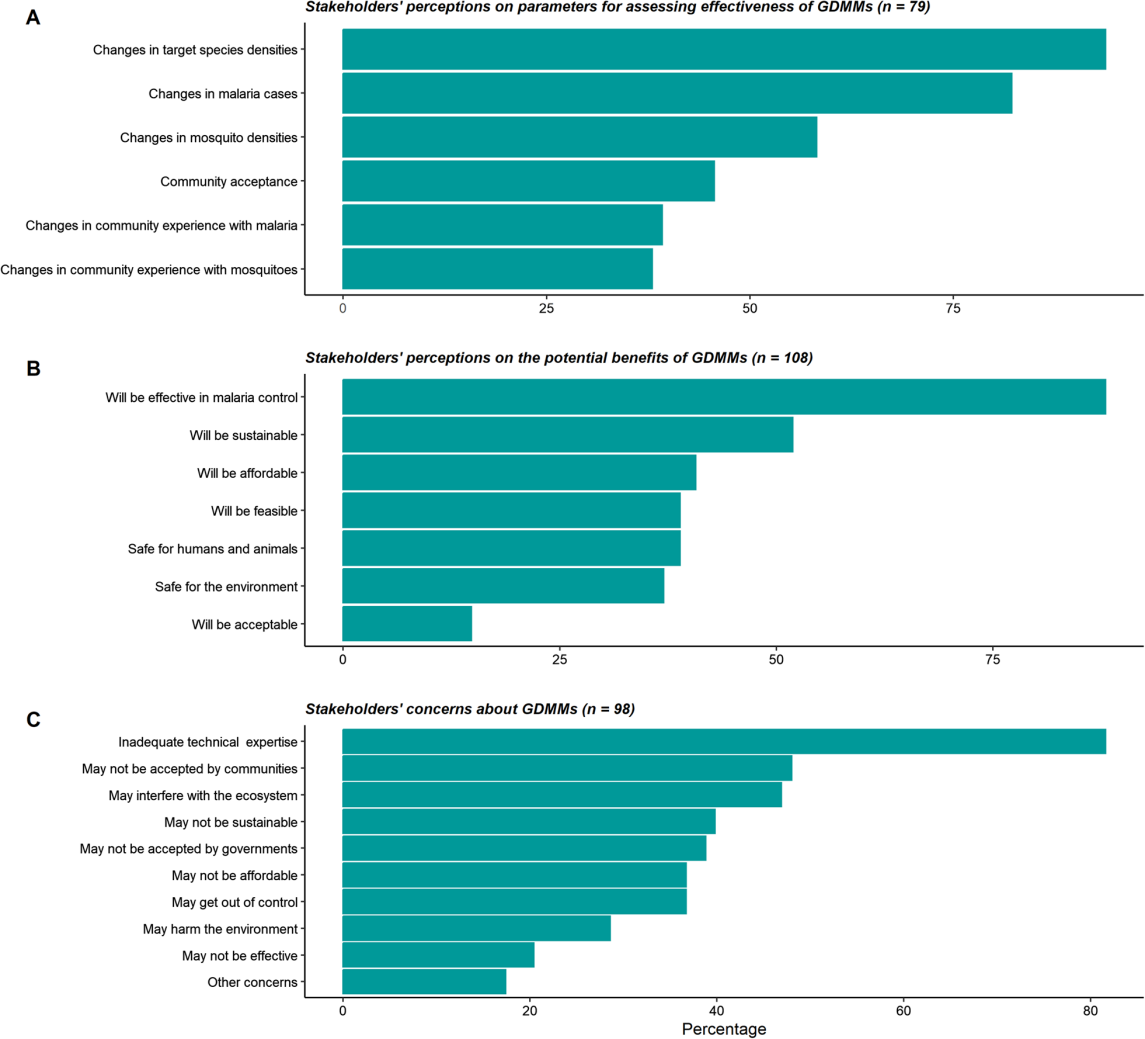
Stakeholders’ perceptions of GDMMs for malaria control and elimination; 3A: Perceptions on which parameters should be used to assess effectiveness of GDMMs; 3B: Perceptions on the potential benefits of GDMMs; 3C: Perceptions on key concerns about GDMMs

A great deal of interest on GDMMs was expressed among all stakeholder groups, particularly after receiving the basic explanation from the discussion facilitators. Most participants expressed their reliance on evidence-based information and expertise from scientists to form their opinions on the potential risks and benefits of GDMMs. Among the 138 respondents who were aware of GDMMs, 57.2% (n = 79) believed that effectiveness of GDMMs could be assessed by monitoring indicators such as reduction in overall mosquito densities, decline in densities of targeted species, decline in malaria cases and community acceptance (Fig. 3A). One participant emphasized the importance of having a strong evidence base to support the technology:

*“For any kind of gene drive technology or kind of implementation platform, what you need is a good evidence base, from which you can sell the technology, because at the end of the day this is what you need to do. What you need is the strength of evidence and that should speak for itself regardless of what supporters or antagonists speak about.”* (Male scientist).

Despite such sentiments, the participants still discussed the technologies intensely, raising questions about GDMMs effectiveness, especially when integrated with current malaria control strategies. In this regard, the participants recommended that a detailed plan should be developed to inform how GDMMs will be integrated, and how their differential impact will be assessed in the presence of, and in comparisons with currently available tools:

*“We should have clear plans of what entomological and epidemiological endpoints we want to achieve, and what acceptable risks are. We also need to determine if these endpoints* c*an these be achieved by just gene drives, or are we able to achieve them with current tools? We need to be able to confidently say that this technology is going to have additional value to current tools.”* (Female lecturer).

#### Perceived benefits of GDMMs

Participants expressed their optimistic views about GDMMs and highlighted several potential advantages for malaria control. A majority (78.3%, n = 108) of the survey respondents recognized the benefits of GDMMs, with 21.7% (n = 30) reporting that they did not perceive any benefits. Those that deemed GDMMs beneficial emphasized on their potential effectiveness, sustainability, affordability and feasibility (Fig. 3B). These perceived benefits also emerged during the in-depth discussions, where participants expressed their hopes that the GDMMs could transform the fight against malaria. Notably, participants were intrigued that a small number of modified mosquitoes released could potentially lead to substantial reduction or even the elimination of malaria in some settings. According to the participants, GDMMs could contribute to more affordable, sustainable, and environmentally-safe malaria control efforts as this participant said:

*“This technology is fascinating. If there is evidence that it will work as you say, then it could be an end to malaria, as it seems to address some of the major challenges that the current tools are facing. It could also save a lot of money that is going to resolve the challenges that we are currently facing.”* (Male government official).

### Concerns about GDMMs

Concerns about GDMMs were expressed by 71.7% (n = 98) of the respondents, while 28.3% (n = 40) reported no concerns. Major concerns included inadequate technical expertise in the countries, unpredictable community and country-level acceptance, risk of ecosystems imbalance due to introduction of GDMMs, and concerns related to affordability, or sustainability (Fig. 3C). Similar concerns were expressed during the in-depth discussions, where participants emphasized the need for evidence to address or mitigate the risks. Participants emphasized the importance of not blindly accepting new technologies and stressed the need for clear comparisons between the anticipated benefits and potential risks associated with the technologies. Furthermore, participants expressed the need for evidence on how GDMMs would complement the current vector control tools such as ITNs and IRS, and asked about the capacity of GDMMs to address challenges such as insecticide-resistance and multiple malaria vector species. They also inquired how GDMMs could make sense in settings where malaria control efforts are aimed at eliminating the vectors:

*“For gene drives to be successful, the modified mosquitoes have to be persistent in the environment. That goes against the currently available tools which aim to eliminate mosquitoes. Do we stop implementing the current tools in order for gene drives to work? For how long? How long can this technology take for us to realize its benefits?”* (Male scientist).

Participants also expressed their concerns that GDMM research currently focuses on *An. gambiae s.s*, the species that is the focus of most indoor control methods such as ITNs and IRS, and has even been effectively controlled in some localities. They also noted that the species might not be the dominant vector in all localities [42–44]. They wondered whether focusing on this species would be adequate for malaria control efforts and recommended that GDMMs should be developed with specific contexts in mind, to target the main vector species in different settings. The discussions highlighted the importance of tailoring GDMMs to address the multiple malaria vector species in Africa:

*“Anopheles gambiae, the species that is currently being researched, is not the dominant malaria vector in our country, it has even been eliminated in many settings. If this technology is really meant to respond to our country needs, then it must be tailored to respond to our challenges. Species of importance for us are Anopheles arabiensis and Anopheles funestus; any hopes of eliminating malaria will focus on these species.”* (Female lecturer).

The participants, however, acknowledged that many of the expressed concerns could be addressed through field trials in malaria-endemic countries. They recognized that the behaviour of the modified mosquitoes might differ between the laboratory conditions and the natural environment. One participant emphasized the necessity of conducting field trials to address these specific questions and uncertainties surrounding GDMMs:

*“How will we know that this technology works unless it is in the community? We need to take the dive and accept field trials so that we have answers to a lot of these questions. These cannot be answered by lab research.”* (Female scientist).

Regarding the lack of technical expertise, participants discussed their own limited understanding and misconceptions regarding the nature of GDMMs, and the perceived limited knowledge of the technologies among communities in their countries. Concerns about whether African malaria control experts had a thorough understanding of GDMMs were raised, and it was recommended that this be a top goal before consenting to introduce these technologies in their countries:

*“How many of the great minds in the country have critical knowledge of this technology? Who knows the details of how it works, or of when things have gone wrong? We should not be swayed by donor funding to accept something that we are not prepared for. We have priorities with regards to this technology, and we should make sure that those have been met before we say yes to it. Building local expertise is a priority to me.”* (Male lecturer).

Moreover, participants talked about inadequate knowledge of genomics among African scientists, stemming from absence of this topic in the basic science curriculum in schools. Several potential solutions were proposed, such as changing the curriculum in schools to include instruction on the fundamentals of genomics, sponsoring African scientists for hands-on training on the technologies, and building capacity of African regulators and decision-makers to understand and effectively regulate these technologies:

*“It is important to build local capacity. Capacity of those researching the technology, and those with authority to make decision about it. We have to also build our capacity so that we can look at the applications and make decisions with confidence.”* (Male regulator).

The concern over the safety of these technologies was debated widely during the in-depth discussions. Participants emphasized the importance of conducting rigorous safety studies to ensure that the modified mosquitoes do not mutate and become invasive, transmit other infections or cause any unintended harm to humans and the ecosystem. With regards to invasiveness, participants elaborated their fears that the modified mosquitoes could dominate over other mosquitoes, and become difficult to control with current methods, which could result in decline of non-targeted species and cause unintended harm to humans and the ecosystem, as this participant elaborated:

*“My concern is that these mosquitoes could outcompete all others and become super mosquitoes that cannot be controlled. Even if they no longer transmit malaria, they could still transmit other diseases or cause nuisance bites, so to me that is a bigger threat than malaria.”* (Female policy maker).

Participants recommended conducting broad studies on the vectors and biodiversity to establish baseline data for post-implementation surveillance. In the event of adverse events requiring mitigation, participants suggested resorting to aerial insecticide sprays only if no other mitigation plans were available.

### Community and stakeholder engagement

Community and stakeholder engagement (CSE) was among the most discussed themes during the in-depth discussions; participants emphasized the importance of involving all relevant stakeholders extensively in awareness-raising and decision-making activities to ensure successful research and implementation of the technologies. To navigate the governance structures in most African countries, a majority of participants recommended adopting a top-down engagement approach; they suggested to start with national leaders to secure their support for introducing GDMMs, then progressively cascade the engagements to regional, district and grassroots community levels. They argued that this approach would provide a sense of safety and security among communities, knowing that their decision-makers were well informed and in support of the technologies:

*“This is for any new technology, the main stakeholder would be the government officials, and especially the ministry of health, as these will make a decision on whether or not this technology is accepted in the country. Then you make your way down to other leaders until you get to the communities. You cannot go straight to the communities”.* (Male scientist)

Another point of convergence about CSE was on the timing of engagement with the communities where GDMMs would potentially be deployed. The participants recommended conducting community engagement after the technologies have been developed and are ready for open field trials. This was expressed to ensure that significant technical and safety concerns and inquiries have been adequately addressed, as this participant recommended:

*“Public engagement needs to be delayed for now, when there is so much backlash from COVID-19, but also when there are more questions than answers for this technology. Go to the public when you can answer all key questions, otherwise you will harm its progress.”* (Female scientist).

Furthermore, participants emphasized the need to inform communities regarding how these technologies align with their needs, and how they can contribute. They stressed the significance of incorporating the views and needs of the communities during its development and research. One participant highlighted the importance of considering the relevance of GDMMs to the people before engaging with them:

*“Before going to the communities about this technology, it is important to sit back and think, how does it meet the needs of the people? How this technology is delivered to the people needs to be informed by the people themselves.”* (Male scientist).

### Regulatory issues relevant to GDMMs

Regarding regulation of GDMMs, 41.1% (n = 74) of the survey respondents reported awareness of regulations related to GDMMs research, while 31.7% (n = 57) reported awareness of regulations concerning the implementation of the technology (Table 5). However, it was unclear whether the respondents fully understood the contents of these regulations. During in-depth discussions, it became evident that most participants had limited knowledge of the underlying issues around GDMMs-related regulations for research or implementation. When asked to outline specific regulatory questions around GDMMs, a majority of the participants admitted their lack of awareness. Nonetheless, some participants, particularly regulators, mentioned that although there are currently no regulations specific for GDMMs, existing guidelines in the agricultural sector pertaining to genetically modified organisms (GMO) could be reviewed and adapted to address GDMMs requirements:

**Table 5:** Awareness of regulations governing GDMMs among survey respondents

Category	Responses	n (%)
Regulations related to gene drives research for malaria control (n = 74)^a^	Regulations on ethical conduct	70 (94.6%)
	Regulations on safety	64 (86.5%)
	Regulations on confinement and field trial	58 (78.4%)
	Regulations on ecosystems safety	58 (78.4%)
	Regulations on cross-border/movement	54 (73.0%)
Regulations related to implementing gene drives for malaria control (n = 57)^a^	Regulations on ethical conduct	50 (87.7%)
	Regulations on cross-border/movement	46 (80.7%)
	Regulations on ecosystems safety	41 (71.9%)
	Safety	44 (77.2%)
	Regulations on confinement and field trial	40 (70.2%)

^a^Percentages add up to more than 100% due to multiple selections

*“We can start with regulations on GMO-agricultural products, that can inform research into these technologies, then the regulations can be updated as evidence increases.”* (Male scientist).

Regulators who participated in the in-depth discussions explained that the existing regulations for genetically modified organisms in Africa primarily focus on environmental protection, with no specific regulations addressing genetic modification technologies in the health sector. They suggested that the existing regulations, such as the Cartagena Protocol on Biosafety and the United Nations Convention on Biological Diversity (CBD) [19, 45], could offer detailed guidance on how to regulate technologies like GDMMs. They further recommended that specific countries considering to use these technologies should base their decisions on their specific circumstances and needs:

*“You have to approach it from the country perspective. You see the Cartagena protocol is coming out with new ideas towards rolling out gene drives, and other emerging technologies like synthetic biology, or gene editing. So countries must adopt this, they must have this in their own laws and regulations. You can never be an island on your own. And even though they are not going to adopt it, they need to have very good regulatory structures that should protect the country in case the neighbors adapt it.”* (Male regulator).

Additionally, regulators suggested that African regional organizations, such as the East African Community (EAC), or Economic Community of West African States (ECOWAS) should make efforts to establish, or harmonize the relevant policies and regulations for GDMMs at regional and continental levels. They commended the ongoing efforts of key organizations, such as the Africa Union Development Agency’s New Partnership for Africa’s Development (AUDA-NEPAD) and encouraged learning from their experience as this regulator said:

*“NEPAD has been playing a key role in bringing harmonization in countries. They have started with western Africa. And in east Africa, we have the East Africa Community, and we also have the East Africa Science and Technology in Rwanda, that has been trying very hard to bring harmonization in the region.”* (Male regulator).

### Support for GDMMs for malaria control and elimination

Despite the concerns raised, most (92.9%, n = 128) of the survey respondents expressed support for field trials or implementation of GDMMs for malaria control in their respective countries. Only 7.2% (n = 10) did not support or were undecided about GDMMs (Fig. 2D). This support was also evident during the in-depth discussions, where participants emphasized the potential of GDMMs to complement existing malaria control efforts. However, there were divergent opinions among the stakeholder groups regarding the preferred approach for GDMMs. Most participants, across the different stakeholder groups, deemed the population suppression approach more likely to be accepted by communities in endemic settings. They argued that people generally associate high densities of mosquitoes with malaria, hence suppression of mosquito population will more likely readily gain acceptance:

*“If you are to consider needs of the affected communities, population suppression would be the best approach, because people know what they have been through. They see a threat, they kill. Mosquitoes are sort of like snakes; you know there are some snakes that don’t bite, but when you come from a setting with poisonous snakes, when you see one you never stop to consider ethics of killing it, because the moment you stop it kills you, so you just kill it. So it is the same with mosquitoes, you cannot expect people who have lost their loved ones to say ‘*let’s conserve the ecology’, *they will just want to see the threat gone.”* (Female reporter).

However, some participants raised concerns about the potential harm to the ecosystem if mosquitoes were entirely eradicated, as mosquitoes also serve certain ecological purposes. These participants preferred the population replacement approach, which eliminates the parasite while maintaining the mosquito populations, thus preserving the ecosystem:

*“I would very much love population replacement so you are actually replacing the fact that the vector will no longer transmit the parasite. For me this is more sensible, it will make the technology somehow balanced.”* (Male scientist).

On the other hand, there were also participants, particularly scientists, who proposed a sequential combination of the two approaches. They suggested starting with a population replacement approach to eliminate the parasite, and subsequently deploying population suppression technologies to reduce vector densities. According to these participants, this approach would allow careful monitoring and provide data on the benefits and risks of both approaches. It would also be useful as a means of mitigating potential risks:

*“I think maybe we start first with population replacement so that the vectors cannot transmit the parasite, and of course if the vectors cannot transmit the parasite, we can now go on slowly but carefully with population suppression to reduce the densities of the populations.”* (Male scientist).

While expressing support for GDMMs, participants emphasized the importance of country-specific evidence regarding their effectiveness, safety and applicability in different sub-Saharan African settings where they would potentially be deployed. They stressed the need for robust scientific evidence on various aspects, such as the fitness and survival of modified mosquitoes in different climatic conditions, their ability to compete wild vector populations, and the acceptance of the technology by communities:

*“Evidence is the most important need here, strong scientific evidence of how it will respond to the situations that different malaria endemic settings are facing… Will the modified mosquitoes survive in different climatic conditions in malaria-endemic Africa? Will they outcompete the wild vectors? Will the communities accept it? This evidence will eventually determine the support for the technology.”* (Female scientist).

In line with this, participants recommended conducting detailed studies, including field trials and mathematical modelling, to generate evidence on how GDMMs can fit within integrated vector management strategies. They also emphasized the need for cost-benefit analyses to compare the GDMM approaches with other malaria interventions and assess their potential when used alone or in combination with existing interventions.

## Discussion

To our knowledge, this is the first comprehensive multi-country investigation of the perspectives and recommendations of stakeholders across malaria-endemic Africa regarding the potential of GDMMs for improving malaria control and elimination efforts in the region. The stakeholders involved in this study comprised of key decision-makers in African malaria control sphere, making their insights and perspectives invaluable in setting the research agenda and prospects for future utilization of GDMMs in Africa.

**Fig. 4:**
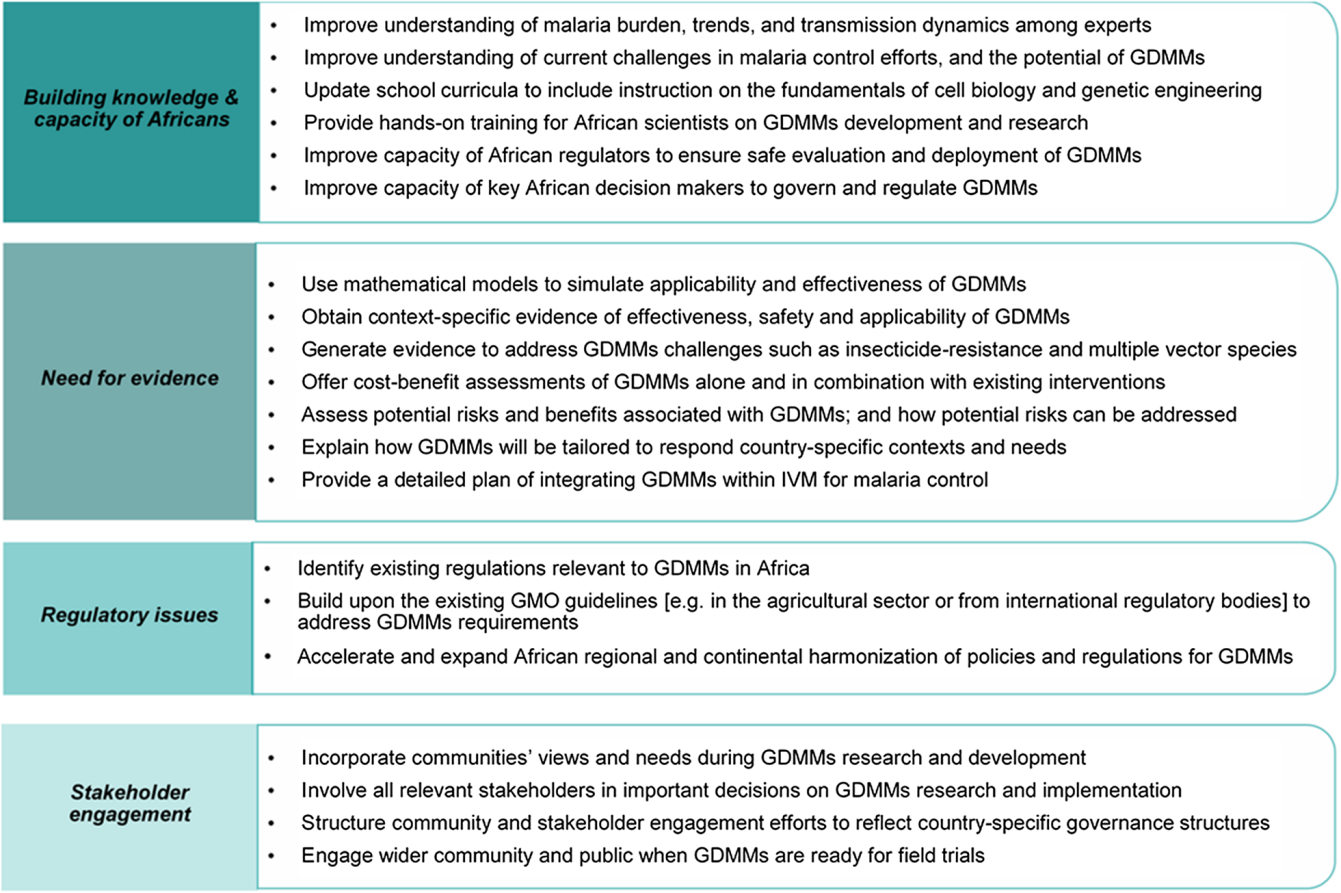
Summary recommendations of key African stakeholders on GDMMs

While previous studies have reported a high level of knowledge about malaria symptoms, prevention, and treatment among different stakeholder groups across Africa [46–49], this study revealed some gaps in understanding of the current malaria situation, especially among key decision makers in the continent. Nearly half of the survey respondents in this study could not indicate reasonable estimates of the malaria burden in their countries. Additionally, a significant proportion of the participants had limited knowledge of dominant malaria vectors in their countries. In several cases, the selected species were either not major vectors anywhere in Africa, or they were not dominant in the countries the participants represented [50]. This knowledge gap could lead to exaggeration or underestimation of possible benefits associated with GDMMs in the continent, potentially affecting the perceived need for the technologies. Addressing this knowledge gap is a critical prerequisite for alternative strategies such as GDMMs, especially considering their species-specific nature. Moreover, according to the health belief model (HBM), awareness of a magnitude and severity of a problem directly influences the perception of the potential benefits of a solution [51]. Therefore, engagement strategies for GDMMs should include a focus on improving the understanding of key stakeholder groups regarding both the malaria situation and the technology itself; as well as more specifically, the knowledge about dominant local vectors and existing control challenges (Fig. 4).

The survey respondents demonstrated relatively high levels of awareness about GDMMs, which can be attributed to extensive discussions on this topic in various communication forums. This contrasts with previous studies conducted in Africa, which indicated relatively low levels of awareness of similar technologies among stakeholders in Mali [52], Nigeria [53] and Tanzania [54, 55]. The difference may be attributable, at least in part, to increasing internet coverage and expanding reach of social media. Furthermore, the participants in this study, mostly researchers and academics, were more likely to be informed about these technologies compared to participants in previous studies.

Unfortunately, the high levels of awareness of GDMMs was clouded by a limited technical understanding, as demonstrated by the inability of most respondents to explain the biological or genetic basis underpinning GDMMs for malaria control, and to distinguish them from other mosquito-modification technologies. Nearly three-quarters of the survey respondents reported no knowledge of how GDMMs work in malaria control. This was unexpected, especially among scientists involved in malaria research. Although there have been significant scientific communications on GDMMs over the past decade, it is likely that much of this information has been very generalized, with more mechanistic discussions limited to those directly involved in research on GDMMs. This suggests the need for better-structured programmes that provide graduated levels of technical knowledge to the satisfaction of different stakeholder groups.

At the time of conducting this study, there had been no GDMMs research activities going on in any African nation. Preliminary investigations being carried out by organizations like Target Malaria in Burkina Faso, Ghana, Uganda, and Mali, the Transmission Zero group in Tanzania, and the University of California Malaria Initiative in Sao Tome and Principe had centered on establishing technical capabilities and collecting baseline entomological data. Research on GDMMs at the time was primarily conducted within academic and research institutions situated in Europe and North America. It was, therefore, surprising that about a third of the survey respondents reported being aware of ongoing GDMMs research in their countries, spanning from laboratory-based research to large field trials. It became apparent during the in-depth discussions that the participants often confused GDMMs with other genetic biocontrol technologies, and that the confusion was likely due to the limited understanding of the difference between GDMMs and the other biocontrol technologies with similar goals. Examples included the Sterile Insect Technique researched in South Africa [56], or investigations of (non-gene drive-containing) genetically modified mosquitoes ongoing in Burkina Faso [57] at the time of this study. Moreover, there were also investigations into the potential of GMMs for malaria control in in Mali and Uganda [18] at the time of this study, although it was not clear what phase of research was going on. This finding suggests the urgent need for simplified but accurate information to lay communities, about GDMMs and how they differ from other mosquito modification technologies (Fig. 4).

Others have proposed that effective communication on technologies such as gene drives, requires context-specific knowledge that identifies the problem’s meaning in a specific setting and how the technologies can address it [29, 58]. This was clearly reflected in this study, particularly during the in-depth discussions. Participants emphasized the need to generate country-relevant evidence on the effectiveness and safety of GDMMs, considering the malaria situation, dominant vectors and existing challenges. This evidence could demonstrate applicability, effectiveness and safety of GDMMs (Fig. 4).

The limited knowledge of regulations to guide research and implementation of GDMMs, as observed in both the survey and in-depth discussions, is not surprising given the novelty of this technology. However valuable insights were gleaned from the in-depth discussions, particularly on how countries can adapt existing international conventions and protocols such as the Cartagena Protocol on Biosafety to develop regulations for governing GDMMs, regardless of whether the countries decide to deploy the technologies or not. The participants also highlighted the importance of harmonizing regulatory frameworks across Africa, and identified organizations that facilitate these efforts, such as AUDA-NEPAD and African regional organizations [59]. The participants acknowledged that no public health-based regulations aimed at gene drives currently exist, and recommended that these should be clarified. The relevance of multisectoral regulations has also been recommended by National Academies of Sciences, Engineering and Medicine (NASEM), to ensure both public health and environmental safety [16].

Community and stakeholder engagement emerged as highly debated aspects of GDMMs, with a general consensus among the study participants on its importance in ensuring technology acceptance and success. This sentiment aligns with the broader role of CSE in obtaining technology support and acceptance [16, 60]. The participants in this study, most of whom worked in academia or government, recommended tailoring CSE efforts to fit within specific countries’ governance structures (Fig. 4). In this regard, they emphasized the need for top-down approach engagement in many African countries, as this aligns with their governance structures. The importance of involving local stakeholders, and of adapting CSE efforts to local context has also been recommended in previous studies across Africa [52–55], as well as in guidelines from NASEM and WHO [16, 60].

Despite identifying multiple ongoing needs, the participants highly supported a phased approach to research and implementation of GDMMs in their countries, even as additional evidence on safety, applicability, and effectiveness is gathered. These findings counter dominant voices from previous studies expressing that similar technologies may not be acceptable in Africa [15, 53, 55]. A majority of the survey respondents and in-depth discussion participants expressed their support for staged field releases and eventual deployment of GDMMs in their countries, acknowledging the potential of GDMMs in improving malaria control and elimination efforts. These findings align with previous studies in Africa, where community members in Mali and Tanzania, as well as research scientists in Nigeria expressed their support for modified mosquitoes technologies, subject to evidence of safety and effectiveness [52–55]. The participants in this study provided specific recommendations for increasing the critical mass of local expertise, generating local evidence, considering country-specific contexts in technologies’ development and CSE efforts, and adapting regulations to account for specific country-situations (Fig. 4). Addressing these recommendations thoroughly would improve the options for successful implementation of GDMMs as public health tools in Africa.

### Limitations

This study was conducted between 2020 and 2022, during COVID-19 era, which restricted the mode of discussions to be done on online platforms like Zoom. As a result, direct participation and contribution of the participants were limited. Additionally, there was uneven representation of countries in the survey and in-depth discussions, with participants ranging from one to more than 20 representatives per country (Fig. 1). Furthermore, certain stakeholder groups such as regulators and media persons were underrepresented compared to the other groups (Table 1), making it difficult to draw a meaningful comparison between the groups. It is also important to note that the stakeholders participating in this study were not community representatives, which means that, the community-based recommendations provided may not necessarily reflect the views of the actual communities in malaria endemic settings. Moreover, in Tanzania the in-depth discussions were conducted in-person due to lifting up of COVID-19 restrictions, which resulted in greater participation from all the stakeholder groups compared to the other countries. Despite these limitations, the study has generated valuable baseline data on the level of awareness among different stakeholder groups on GDMMs, and the existing gaps in the research and deployment of GDMMs for malaria control in Africa. It also sheds light on the initial perceptions and recommendations of key African decision makers regarding the potential of utilization of GDMMs to benefit malaria control efforts in the region.

## Conclusion

This study amplifies African stakeholders’ voices on important preconditions for GDMM implementation in addressing malaria in Africa, encompassing technical expertise expansion, local evidence generation on safety, applicability and effectiveness of GDMMs, and context-specific regulations. Despite stakeholder awareness of GDMMs, at the time of this study their understanding of its malaria control applications remained limited. Nevertheless, the majority supported GDMM trials or implementation. The stakeholders’ recommendations offer guidance to ensure the safety and efficacy of GDMMs in malaria control, respecting the political, governance, and cultural dynamics in malaria-endemic countries in Africa.

## Data Availability

All data for this study will be available upon request.
